# Robust filtering of thin‐slice reconstructions improves lacunar stroke detection in CT perfusion imaging

**DOI:** 10.1002/mp.70271

**Published:** 2026-01-14

**Authors:** Joris Vromans, Edwin Bennink, Jan Willem Dankbaar, Birgitta K. Velthuis, Hugo W. A. M. de Jong

**Affiliations:** ^1^ Department of Radiology and Nuclear Medicine University Medical Center Utrecht Utrecht The Netherlands

**Keywords:** CT perfusion, denoising, lacunar stroke, noise filtering, thin‐slice reconstructions

## Abstract

**Background:**

The detection of lacunar strokes in CT perfusion (CTP) imaging may be improved by reducing the reconstructed CTP slice thickness. However, this increases noise, necessitating the use of robust noise filters.

**Purpose:**

To Investigate the impact of slice thickness and three state‐of‐the‐art noise filters on the detection of lacunar strokes of varying sizes.

**Methods:**

Artificial spherical lacunar infarcts (diameter: 5, 7, and 10 mm) were added to 40 thin slice CTP acquisitions. The performance of time‐intensity profile guided filtering, temporal‐average guided filtering, and a U‐Net based filter were compared based on infarct detectability in cerebral blood volume maps of thick‐ (4.9 mm) and thin‐slice (0.7 mm) reconstructions. One observer evaluated the maps for each combination of filter and slice thickness. A second observer repeated the evaluation for the two best performing combinations. F1‐score, contrast‐to‐noise ratio, and observer confidence were calculated for every combination of filter, slice thickness, and observer.

**Results:**

The highest F1‐scores were achieved with the temporal‐average guided filter (thin slices: 0.70, thick slices: 0.49). The other filters did not exceed an F1‐score of 0.36 on either thin‐ or thick slices. Thin‐slice temporal‐average guided filtering also achieved the highest contrast‐to‐noise ratio and observer confidence, which resulted in a sensitivity of 10% for 5 mm, 73% for 7 mm, and 75% for 10 mm diameter lacunar infarcts.

**Conclusions:**

This study demonstrates that robust noise filtering, particularly temporal‐average guided filtering, enables improved detection of lacunar strokes (≥7 mm) in CTP imaging using thin‐slice reconstructions.

## 1 INTRODUCTION

Lacunar strokes are caused by small vessel occlusions that result in small ischemic regions located in noncortical areas, mostly in the basal ganglia, and are usually smaller than 15 mm in diameter.^1^, ^2^ They account for up to 25% of ischemic strokes. Of all patients suffering from lacunar stroke, 20% experiences a recurrent cerebrovascular event, and 30% will be functionally impaired at a 5‐year follow‐up.^3^, ^4^ For these reasons, detection of lacunar stroke is important. Although MRI with diffusion‐weighted imaging (DWI) is the gold standard to detect lacunar infarcts it is less routinely available in the acute setting than CT.

CT perfusion (CTP) is a highly sensitive method to detect and assess ischemic infarcts from large vessel occlusions and to guide treatment decision‐making.^5^, ^6^ It provides quantitative maps that help differentiate between the irreversibly damaged infarct core, where tissue viability is lost, and the surrounding salvageable penumbra, where timely reperfusion can prevent infarction. However, the images contain relatively high noise that prevents straightforward analysis of high resolution thin‐slice images. Therefore, CTP images are commonly reconstructed to a slice thickness of 5 mm to reduce the noise and computation time. As a consequence, detectability of lacunar strokes is compromised by partial volume effect. This contributes to missed lacunar strokes (sensitivity: 0%–62.5%, specificity: 20%–100%) on the perfusion maps in the acute phase.^7^

CTP noise filters attempt to reduce the noise while retaining the signal, and are vital to proper thin‐slice analysis. Promising approaches to cope with CTP noise include state‐of‐the‐art convolutional neural networks, such as U‐Net, and bilateral filters, such as time‐intensity profile similarity filtering (TIPS) and temporal‐average (tAvg) guided filtering.^8^, ^9^, ^10^ The U‐Net denoising method by Wu et al. involves numerous parameters that can be optimized for improved denoising performance. In contrast, the bilateral filters only use two parameters. The filters average voxels based on intensity similarity and spatial distance. TIPS evaluates the entire time‐attenuation‐curve (TAC) for intensity similarity, allowing it to take temporal dynamics into account. However, it remains sensitive to noise, as noise directly impacts TAC similarity measurements. Temporal‐average guided filtering mitigates this sensitivity by comparing TAC means instead, which enhances robustness to noise at the expense of losing information about the temporal dynamics.

To evaluate the practical implications of these filter differences and the image reconstruction thickness, we compared the detectability of lacunar strokes on high‐noise thick‐ and thin‐slice reconstructions for three state‐of‐the‐art CTP noise filters using clinical CTP images with artificially added lacunar infarcts.

## 2 METHODS

### 2.1 Artificial lacunar infarcts

**FIGURE 1:**
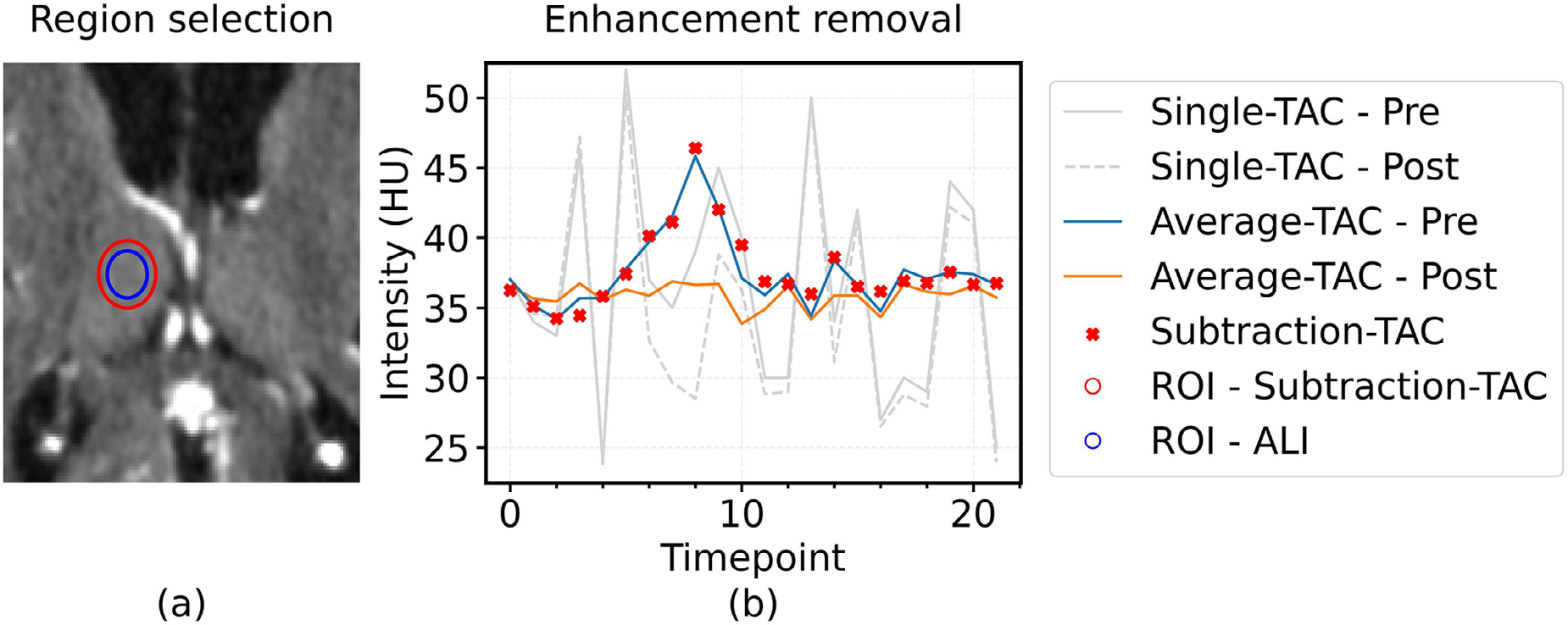
Creation of artificial lacunar infarcts (ALIs). (a) First, a sphere is selected as an ALI region (blue circle, in this case 7 mm diameter). (b) Second, the average enhancement from voxels within the 10 mm sphere (red circle) is subtracted from the voxels within the ALI region, leaving time‐attenuation curves without enhancement but with realistic and natural noise. ALI: Artificial lacunar infarct; ROI: region of interest; TAC: time‐attenuation curve.

**FIGURE 2:**
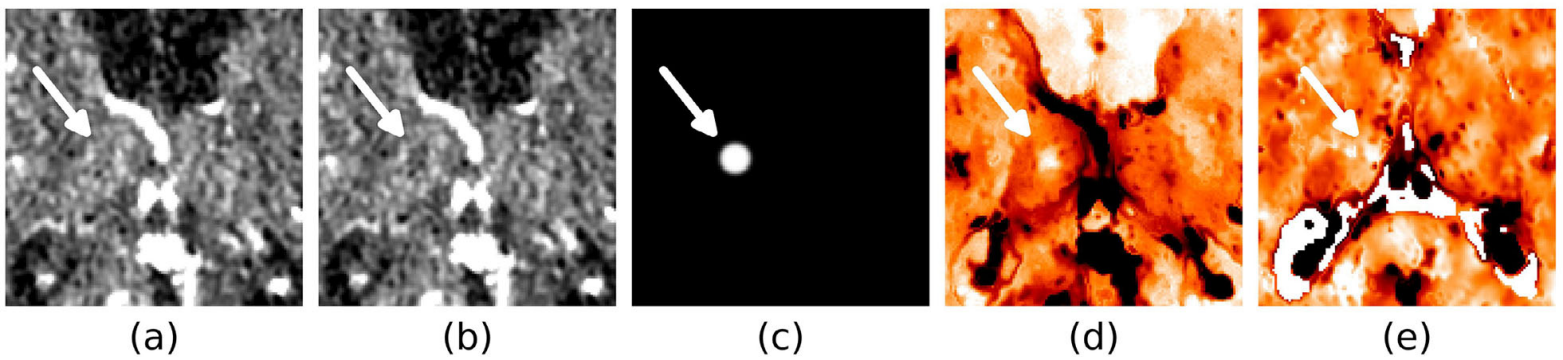
Visualization of an artificial lacunar infarct (ALI) and real infarct, both located in the right thalamus. (a) Contrast‐peak frame without an ALI. (b) Contrast‐peak frame with an ALI. (c) Subtracted contrast (white is 10.7 HU). (d) CBV map with ALI. (e) CBV map with real infarct (different patient).

The availability of CTPs with confirmed lacunar strokes is restricted due to limited timely (within 24 h) follow‐up with MRI. This dataset limitation was overcome by creating artificial lacunar infarcts (ALIs) that also can be used as gold standard facilitating performance assessment. ALI locations included the nucleus caudate, capsula interna, putamen, thalamus, and pons, which are structures where lacunar infarcts are frequently found. On such a location, the iodine contrast enhancement within an ALI‐sphere with a diameter of 5, 7, or 10 mm was removed in a clinical CTP source dataset, mimicking absent cerebral blood volume (CBV), that is, the apparent fraction of blood in brain tissue (mL/100 g), in a lacunar infarct core (Figure 1a). To remove the contrast enhancement in a realistic manner, the mean enhancement curve within a 5 mm radius is subtracted from all voxels in the ALI‐sphere, retaining only the background CT intensity and the noise characteristics (Figures 1b and 2a–c). Voxels with a temporal average CT intensity deviating more than two standard deviations from the mean were considered different tissue and were excluded from the ALI‐sphere and 5 mm radius mean enhancement curve derivation.

**FIGURE 3:**
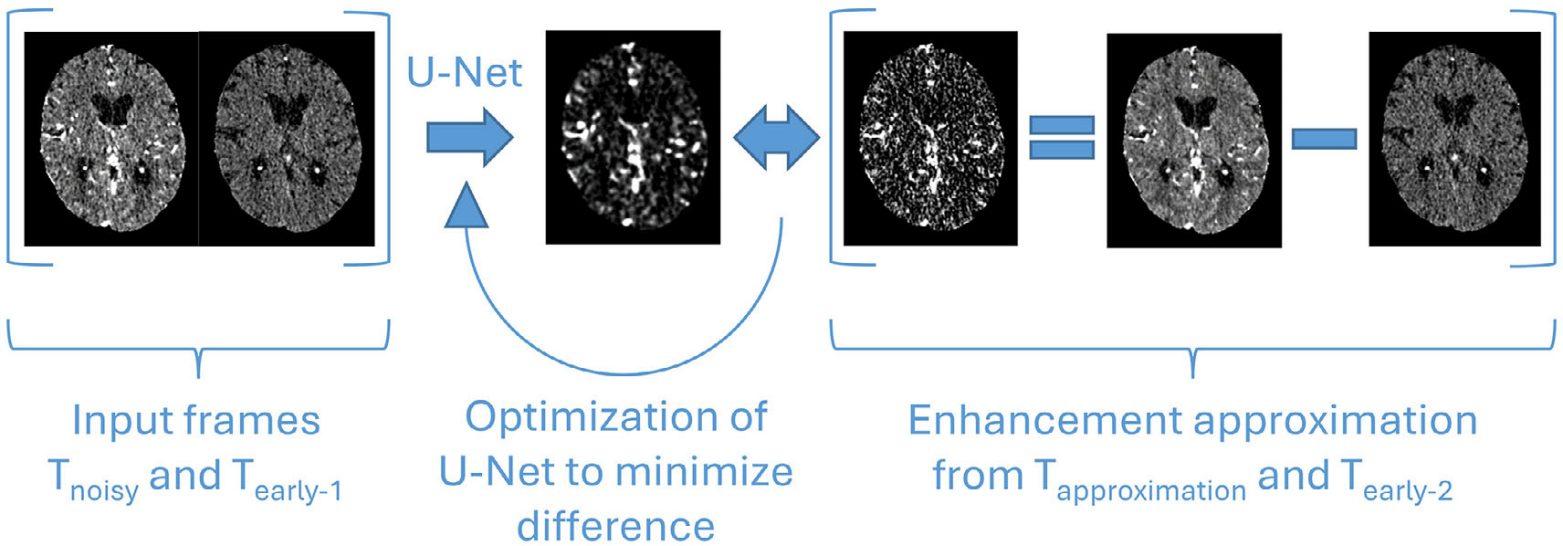
CTP denoising methodology by Wu et al. On the left side, a timeframe (T_noisy_) and an early timeframe (T_early‐1_) enter the network that subsequently predicts the contrast enhancement of T_noisy_. On the right side, a contrast enhancement target is made by subtracting a different early timeframe (T_early‐2_) from the approximation of T_noisy_ (T_approximation_). T_approximation_ is another noise realization of T_noisy_, made by averaging the preceding and succeeding timeframes.

Two neuroradiologists (both with >15 years of experience) confirmed that the ALIs on the CBV maps (Figure 2d) resembled real lacunar infarcts (Figure 2e). Please note that following figures about ALIs (Figures 3, 5, and 6) will show the same representative case as shown in Figures 1 and 2. CBV maps are visualized using the colormap that the radiologists preferred for ALI detection, giving low CBV values a brighter color.

### 2.2 Dataset

A subset of 167 CTP scans from the ENCLOSE study were included. The aim of the ENCLOSE study was to improve the detection of small volume infarcts and thromboembolic sources, and predict recurrent ischemic stroke. The inclusion criteria of ENCLOSE were: patients over 18 years old, stroke onset time less than 9 h, clinical diagnosis of acute ischemic stroke or transient ischemic attack, and informed consent.^11^ The subset was defined by two criteria added to reduce heterogeneity: (1) only data from a spectral CT scanner was used (IQon Spectral CT, Philips Healthcare), and (2) the original acquisition of thin slices (0.7 mm) had to be available. All scans were acquired at 120 kVp and 35 mAs. Thick slices were created by averaging seven consecutive slices, resulting in a slice spacing of 4.9 mm.

#### 2.2.1 Dataset split

The CTP subset (*N* = 167) was divided into test (*N* = 16), validation (*N* = 21), and training (*N* = 130) sets (Table 1). The 16 CTPs of the test set were chosen based on the absence of identifiable ischemia on CTP according to the interpreting radiologist in the acute phase and presence of a gold standard follow‐up MRI. The follow‐up MRIs were used to check whether a potential false positive ALI detection was not a real lacunar infarct. Based on the 16 CTP sets, a total of 50 CTPs were produced to form the final test set for the observer study: 10 cases were selected as control (i.e., without infarcts), and 40 ALIs were placed across all 16 CTPs resulting in a total of 40 3D sets with unique ALIs. Ten of the ALIs had a diameter of 5 mm, 20 had a diameter of 7 mm, and 10 had a diameter of 10 mm, representing a range of lacunar infarct sizes near the detection limit. The ratio between control cases and ALI cases represents the ratio of infarcts (302) and transient ischemic attacks (68) in the ENCLOSE study.^12^ Several measures were taken to reduce bias in the observer study, including: (1) anonymization of both methods and scans, (2) image mirroring, (3) providing five smaller subsets for each method with balanced distributions of infarct size and location, and only unique CTPs, and lastly (4) ordering the subsets to alternate between methods and to separate similar locations. The 21 CTPs of the validation set were chosen from the remaining 151 CTP based on a large area with normal perfusion on CTP. The validation set was augmented by placing 100 ALIs with a diameter of 5 mm in various locations, resulting in a total of 100 2D cases with one ALI each. The remaining 130 3D CTPs (training set) were used for optimization tasks which did not require ALIs.

**TABLE 1:** Overview of the dataset splits and their use cases. CTP: CT perfusion; ALI: Artificial lacunar infarct.

Split	Inclusion criteria	Number of CTPs	Augmentation	Number of cases	Usage
Test	1. No ischemia on CTP 2. Follow‐up MRI available	16	1. 40 ALI insertions 2. 10 controls without ALI	50	Clinical evaluation: 1. Detection performance per method
Validation	1. Large area of normal perfusion on CTP	21	1. 100 ALI insertions 2. 2D slice selection 3. ALI marking	100	Technical evaluation: 1. 2D versus 3D U‐Net 2. Distance sigma
Training	–	130	–	130	Automatic optimization: 1. U‐Net 2. Range sigma

### 2.3 CTP processing

The first preprocessing step corrected for rotation and translation with an in‐house developed method that used a groupwise optimization approach.^13^ Next, thresholding, filling, and connected component analysis of the CTP temporal average was used to create a brain mask and remove the skull. The arterial input functions and venous output functions were automatically estimated from the registered thin‐slice CTPs. Thick slices were derived after the preprocessing steps. After filtering, CBV maps were estimated using a non‐linear regression model.^14^

### 2.4 Denoising methods

Three CTP noise filters were compared based on their performance on thick‐ and thin‐slice reconstructions. These filters were: (1) U‐Net, (2) time‐intensity profile similarity filtering (TIPS), and (3) temporal‐average guided filtering (tAvg).^8^, ^9^, ^10^ All filters were implemented using PyTorch.

#### 2.4.1 U‐Net

U‐Net is one of the most widely used deep learning networks within the medical imaging field, and can be used for various purposes, including image denoising.^15^

##### U‐Net denoising

The Noise2Noise framework provides an optimization target by using pairs of images with identical structural information but different noise realizations: The network attempts to transform one noisy image into the other, which will converge to transforming into a denoised image.^16^ Noise2Noise has become a well‐established method for training image denoising methods in the absence of noise‐free training data. Wu et al. recently used this framework for denoising CTP images and achieved state‐of‐the‐art denoising performance on the ISLES2018 dataset outperforming established methods including TIPS and tensor total variation (TTV).^8^, ^17^, ^18^ They approximated a different noise realization of a timeframe by averaging its preceding and succeeding timeframes. Additional measures were taken to compensate for enhancement bias in the approximation. As Figure 3 describes, the network was trained to predict the contrast enhancement given a CTP timeframe and an early timeframe.

##### U‐Net architecture optimization

The 2D U‐Net implementation was extended to a 3D variant to enable a fair comparison with the 3D bilateral filtering techniques. Furthermore, the standard 3 × 3 convolutional kernels were increased to 5 × 5 (× 5) to account for the higher resolution of the ENCLOSE dataset relative to ISLES2018.^17^, ^18^ Upsampling transpose convolution kernels were adjusted from 3 × 3 to 4 × 4 (× 4).

Each variant of U‐Net was trained twice, once on thick slices and once on thin slices from the training set. Each combination of U‐Net and slice thickness was qualitatively analyzed on the validation set. Two technical experts, with 14 and 17 years of experience in the field of CTP ranked 2D‐ and 3D U‐Net filtered CBV maps based on the discernibility of marked ALIs. This was done on both thick and thin slices, for each of the 100 cases in the validation set.

#### 2.4.2 Bilateral filtering

Bilateral filtering works by combining spatial Gaussian weighting (distance between voxels) with range Gaussian weighting (difference in intensity between voxels), so that only voxels that are nearby and have similar intensities are averaged. This reduces the noise while preserving edges.

In mathematical terms bilateral filtering works as follows; the intensity of each voxels in the CTP (*I_cv_*) is essentially replaced by a weighted average of its neighborhood (*I_nv_*) based on the spatial distance (Δ*d*), intensity difference (Δ*r*), spatial sigma (*σ_d_*), and range sigma (*σ_r_*):

(1) $$I_{cv}=\frac{1}{W_{cv}}\;\cdot \sum_{nv_{i}\;\varepsilon \;kernel}e^{-\frac{{\left(\Delta d_{i}\right)}^{2}}{2\cdot \sigma_{d}^{2}}}\cdot e^{-\frac{{\left(\Delta r_{i}^{2}\right)}^{2}}{2\cdot \sigma_{r}^{2}}}\cdot I_{nv_{i}}$$

The weights in the kernel are normalized to sum to one (*W_cv_*).

##### Time‐intensity profile similarity filtering

**FIGURE 4:**
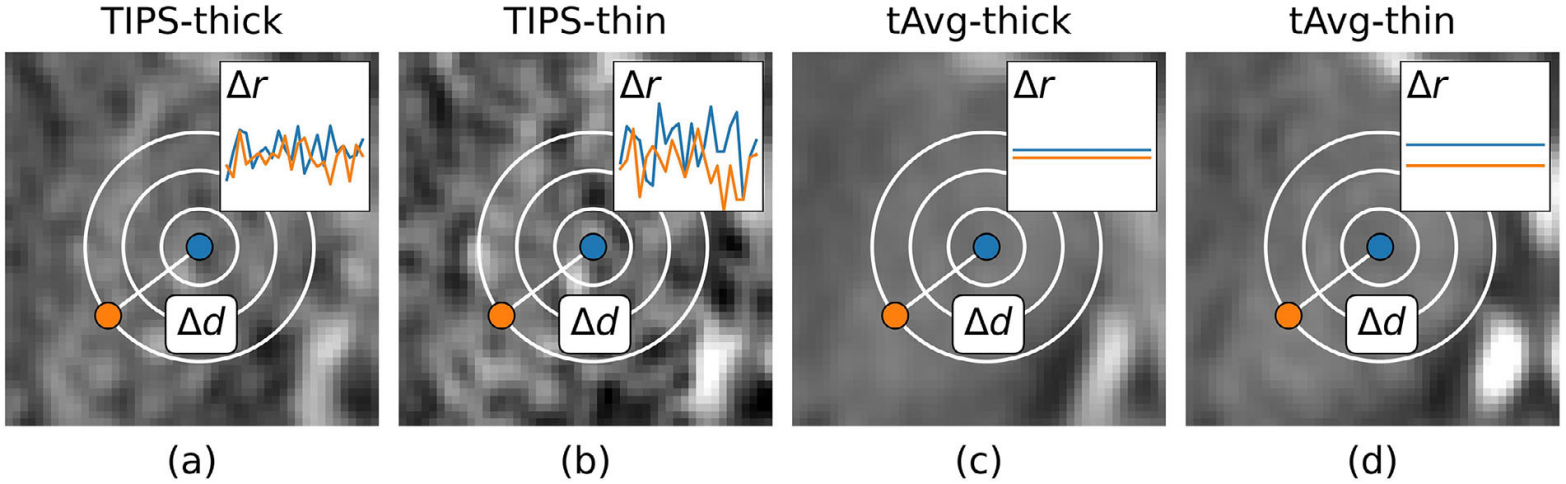
Bilateral filtering. (a,b) TIPS defines the range difference (Δ*r*) as the mean squared difference between the time‐attenuation curves of two voxels. (c,d) tAvg‐guided filtering defines Δ*r* as the squared difference between the means of the time‐attenuation curves. Both filters define the spatial distance (Δ*d*) as the Euclidean distance between two voxels.

Time‐intensity profile similarity (TIPS) filtering is a bilateral filter optimized for denoising CTP images. It uses the mean squared difference between two entire TACs to measure similarity. This means that only voxels with TACs that are very similar in shape will be averaged. This benefits denoising as more information about the voxel is taken into consideration.^9^ However, in thin‐slice reconstructions the signal‐to‐noise ratio of the TACs may become too low for meaningful similarity measurement (Figure 4a,b).

##### Temporal‐average guided bilateral filtering

In contrast to TIPS, the temporal‐average guided filter uses the squared difference in mean of two TACs as a metric for intensity difference (Figure 4c,d). This temporal average is a static, high signal‐to‐noise structural image, which makes the filter robust against noise. However, the temporal average as a guide image has no dynamic information from the time‐attenuation curve such as delay, contrast‐bolus slope, peak‐intensity, and time‐to‐peak, that are key characteristics to distinguish normal‐, infarct core‐, and penumbra tissue during denoising.

##### Kernel optimization

The range sigma (*σ_r_*) weights the difference in intensity between voxels. The range sigma for TIPS (σ_r‐TIPS_) was defined as the mean variance [HU^2^] in time‐intensity profiles (TACs) within the ventricles of the training set.^9^ The range sigma for the temporal‐average guided filter (σ_r‐tAvg_) was defined as the variance in intensity [HU^2^] in all temporal average voxels within the ventricles of the training set.

To select an optimal spatial distance weighting, determined by distance sigma (*σ_d_*), results for values of 1, 2, and 3 mm were qualitatively compared for each combination of bilateral filter and slice thickness using the validation set. Two technical experts ranked 1, 2, and 3 mm filtered CBV maps based on the discernibility of marked ALIs. This was done on both thick and thin slices, for each of the 100 cases in the validation set. The filter dimensions were set equal to three times (*σ_d_*).

### 2.5 Denoising method evaluation

The performance of the denoising methods was evaluated on the 50 test set cases. ALIs in CBV maps were used as ground truth to measure observer confidence and detection performance. CBV was chosen as parameter map because ALIs represent infarct cores that are best described by low blood volume. The evaluation process included a qualitative observer study as well as quantitative region of interest measurements.

#### 2.5.1 Qualitative metrics

A neuroradiologist with >15 years of acute ischemic stroke experience (O1) scored the detectability of the ALIs in the test set of 50 detection cases for allcombinations of filter and slice thickness without knowing where or if ALIs were present. Each 3D CBV map was presented together with a 3D temporal‐average map in ITK‐SNAP where the radiologists could freely navigate the axial‐, sagittal‐, and coronal planes.^19^ A second neuroradiologist with >15 years of acute ischemic stroke experience (O_2_) analyzed the two best performing combinations of filter and slice thickness to measure inter‐observer variability. The ALIs were located and annotated according to a Likert scale (certainly‐, probably‐, possibly‐, probably not‐, certainly not‐ a lacunar infarct). The detection results were expressed in sensitivity, precision, and F1‐score. The threshold for dichotomization of the Likert‐scale was selected based on maximization of the F1‐score. The F1‐score was chosen as leading metric because it emphasizes the detection of ALIs while ignoring the influence of true negatives. The best detection method was analyzed in more depth by computing the sensitivity per structure and diameter to identify potential limitations.

#### 2.5.2 Quantitative metrics

The performance of the denoising methods was also quantitatively measured using the contrast‐to‐noise ratio (CNR) of the 40 ALIs in the CBV maps of the augmented test set:

(2) $$\mathrm{CNR}=\frac{\left|\mathrm{CB}V_{\mathrm{ALI}}\;-\;\mathrm{CB}V_{\mathrm{normal}}\right|}{\sigma_{\mathrm{noise}}}\;$$

CBV_ALI_ is the CBV‐value measured inside the ALI volume in a denoised scan and CBV_normal_ is the CBV‐value measured in the same location in the denoised original scan without the ALI. A homogeneous circular area (diameter = 25 voxels) within one of the thalami was selected in‐plane for each slice thickness to measure the noise (σ_noise_) in the CBV maps.

#### 2.5.3 Statistical analysis

The detection performance (F1‐score, CNR) and observer confidence (Likert scores) of the two best performing methods were tested for significance using paired bootstrapping. The results were deemed significant if the 95%‐confidence interval did not include zero.

The Likert scores were adjusted to compute confidence according to: 1.0: certainly‐, 0.5: probably‐, 0.0: possibly‐, 0.5: probably not‐, 1.0: certainly not‐ a lacunar infarct. Here, “certainly” and “certainly not” have the highest confidence and “possibly” the lowest.

## 3 RESULTS

### 3.1 Optimized filter settings

**FIGURE 5:**
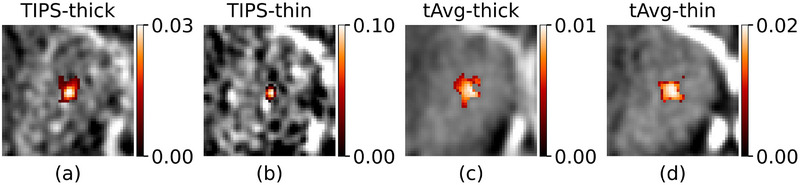
The kernels of the bilateral filters for denoising the center voxel. Although the spatial sigma, *σ_d_*, is the same for all filters, the range kernel (*σ_r_*) and guide image is different which leads in this case to overall smaller kernels for TIPS (a,b) than for tAvg‐guided filtering (c,d).

An example of the kernels of the optimized bilateral filters for a single voxel is shown in Figure 5. The range sigma's for TIPS (σ_r‐TIPS_) derived from ventricle TACs were 37.6 and 59.7 HU^2^ for thick‐ and thin‐slice reconstructions, respectively. The range sigma's for the temporal‐average guided filter (σ_r‐tAvg_) derived from temporal average ventricle voxels were 2.5 and 3.4 HU^2^ for thick‐ and thin‐slice reconstructions, respectively. A spatial sigma (σ_d_) of 2 mm was found to be optimal for all bilateral filter methods, and the technical experts had a preference for 3D U‐Net for both thick‐ and thin‐slice reconstructions over the 2D U‐net.

**FIGURE 6:**
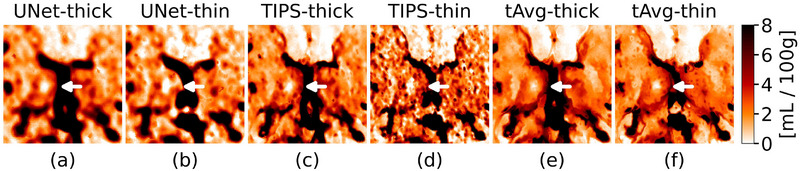
Zoom‐in of CBV maps for each filter and slice thickness. An artificial lacunar infarct (ALI) with a diameter of 7 mm is located in the right thalamus.

The temporal‐average filtered CBV maps show less noise compared to TIPS and U‐Net filtered CBV maps (Figure 6). The thin‐slice maps from TIPS retain a high noise level and the maps from U‐Net appear more blurry. The ALIs on the thin‐slice maps after temporal‐average guided filtering seem to have more contrast than their thick‐slice counterparts.

### 3.2 Denoising method evaluation

#### 3.2.1 Qualitative ALI detection performance

**FIGURE 7:**
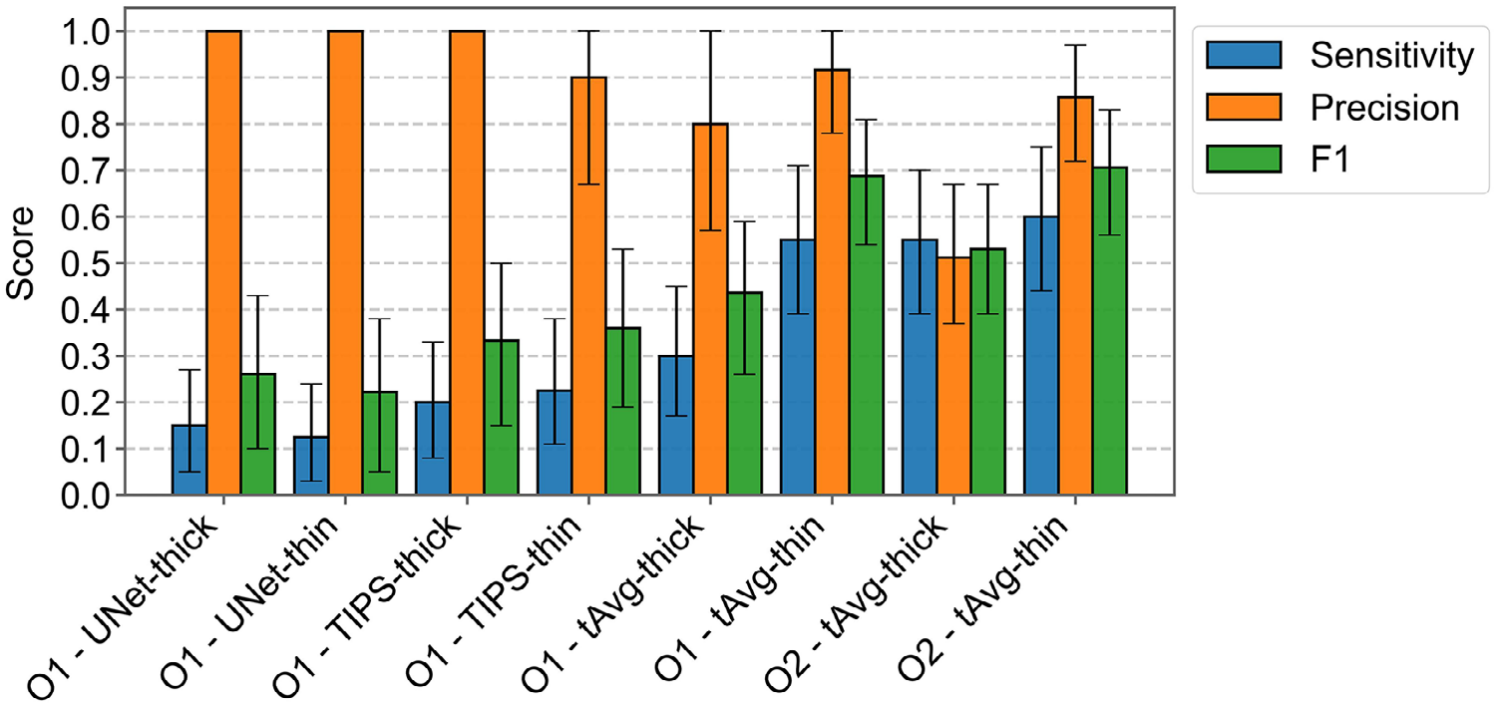
Sensitivity, precision, and F1‐score with 95%‐confidence intervals on the test set for each combination of observer (O1 and O2), filter, and slice thickness.

Figure 7 shows that both observers attained their highest F1‐scores using thin slices with temporal‐average guided filtering (tAvg‐thin; F1‐scores 0.69 and 0.71), while the second best performance was noted for thick slices with temporal‐average guided filtering (tAvg‐thick; F1‐scores 0.44 and 0.53). The improvement in ALI detection by tAvg‐thin over tAvg‐thick was significant for both observers (O1‐ΔCI: [0.09, 0.42]; O2‐ΔCI: [0.02, 0.32]). The difference CIs between the observers with identical methods (tAvg‐thick‐ΔCI: [‐0.26, 0.10]; tAvg‐thin‐ΔCI [‐0.14, 0.10]) included zero and therefore indicated no significant performance differences. U‐Net‐thin and U‐Net‐thick yielded markedly lower F1‐scores (0.22 and 0.26, respectively). The optimal Likert‐scale thresholds for tAvg‐thin were “possibly” for observer 1 and “probably” for observer 2, based on the F1‐score. The observer‐average sensitivity and precision of tAvg‐thin were 0.58 and 0.89, respectively. tAvg‐thick reached an observer‐average sensitivity of 0.43 and precision of 0.69. False positive ALI predictions were checked against the MRI ground truth but none corresponded to real lacunar infarcts.

#### 3.2.2 Qualitative ALI detection observer confidence

**FIGURE 8:**
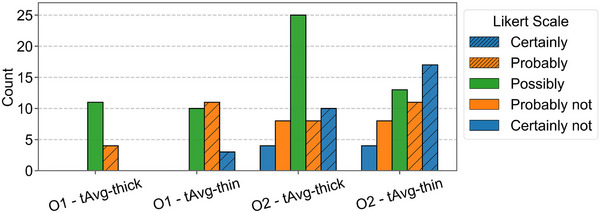
Likert score distributions on the test set for each combination of the tAvg‐guided filter, observer (O), and slice thickness. Inter‐observer variability is visible in the figure, observer 1 (O1) annotated fewer locations and solely used the positive side of the Likert scale. Observer 2 (O2) annotated more locations including non‐infarcts and more often used “certainly”.

In addition to the increase in ALI detection performance for tAvg‐thin, Likert scores containing the terms “probably” and “certainly” were more often selected in thin‐slice reconstructions as can be seen in Figure 8. The improvement in observer confidence by tAvg‐thin over tAvg‐thick was significant for both observers as the difference confidence intervals did not include zero (O1‐ΔCI: [0.17, 0.50]; O2‐ΔCI: [0.01–0.32]).

#### 3.2.3 Qualitative ALI sensitivity

**FIGURE 9:**
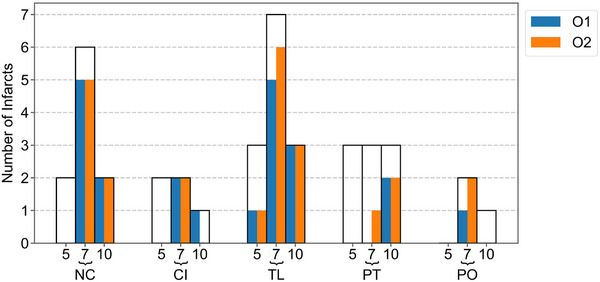
The number of true positives in the test set by structure and diameter (5, 7, and 10 mm) for method tAvg‐thin by each observer (O). (NC: Nucleus caudate, CI: Capsula interna, TL: thalamus, PT: putamen, PO: pons).

Sensitivity for ALIs varied with infarct diameter (Figure 9). For the tAvg‐thin method, the sensitivity for ALIs with a diameter of 7 and 10 mm (73% and 75% detected) was much higher than for 5 mm ALIs (10% detected). Sensitivity for ALIs also varied per anatomical structure (Figure 9). The highest sensitivity was found in the nucleus caudate (70%) and thalamus (73%). The sensitivity was 50% in both the capsula interna and pons. The putamen showed the lowest sensitivity (28%).

#### 3.2.4 Quantitative ALI detection performance

**FIGURE 10:**
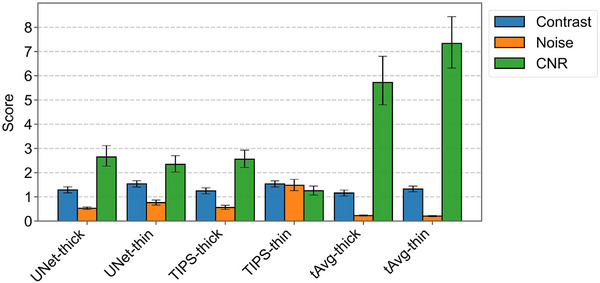
Contrast, noise, and CNR measurements with 95%‐confidence intervals on the test set for each combination of method and slice thickness.

tAvg‐thin achieved the highest contrast‐to‐noise ratio (CNR) at 7.33, which was significantly higher than the second highest, tAvg‐thick, following at 5.72 (ΔCI: [0.84, 2.40]) (Figure 10). All thin‐slice reconstructions showed higher contrast values than thick‐slice reconstructions. tAvg‐thin attained a slightly lower contrast at 1.33 compared to TIPS‐thin (1.53) and UNet‐thin (1.54). TIPS‐thin displayed the most substantial noise increase between thick slices (0.56) and thin slices (1.48), and correspondingly reached the lowest CNR of 1.25 for TIPS‐thin.

## 4 DISCUSSION

The combination of thin‐slice reconstructions and temporal‐average guided bilateral filtering led to the best detectability of lacunar infarcts (F1‐score: 0.70, CNR: 7.33). The significantly better performance over thick slices (F1‐score: 0.49, CNR: 5.72) proves the benefit of increased spatial resolution from thin‐slice reconstructions for the detection of lacunar strokes (O1‐ΔCI: [0.09, 0.42]; O2‐ΔCI: [0.02, 0.32]). The increased spatial resolution also gave the radiologists significantly more confidence in their predictions (O1‐ΔCI: [0.17, 0.50]; O2‐ΔCI: [0.01–0.32]). The radiologists reached a sensitivity of 58% compared to the literature range of 0%–62.5%.^7^

Remarkably, radiologist two used the full spectrum of the Likert scale while radiologist one only used the positive side of the spectrum to indicate ALIs. This discrepancy reflects clinical practice where one radiologist will be more inclined to list the negative findings than another. However, this did not influence the drawn conclusions due to threshold optimization for the Likert scores and use of the F1‐score that disregards true negatives.

The significant performance increase of the temporal‐average guided filter over the other filters (highest F1‐score: 0.36, highest CNR: 2.34) can be attributed to its robustness against noise. It was the only filter that retained a similar noise level in thin slices compared to thick slices while the ALI contrast improved. The other filters, that did not use a robust guide image, showed a lower CNR for thin slices as compared to thick slices.

It is important to note that the temporal average does not contain dynamic information from the TAC such as area under the curve and time‐to‐peak. Filtering with this static guide image would result in a high contrast between grey and white matter, and between infarct core and healthy tissue, but in a lower contrast between penumbra and healthy tissue. A filter with the robustness of a temporal‐average guided filter, but with increased contrast between normal, infarct core, and penumbra tissue in its guide image(s) would benefit stroke detectability even further.

ALIs were used instead of real lacunar infarcts to have a large enough dataset, with a gold standard without possible change in size between admission CT and follow‐up MRI to measure differences between methods with statistical significance.^20^ Although the ALIs resembled infarct core and did not contain any penumbra, the ALIs retained the original noise characteristics and were very similar to real lacunar infarcts.

A few factors have led to increased detection difficulty compared to the previous lacunar stroke detection studies. Firstly, although lacunar infarcts have a typical size between 3 and 15 mm with an upper limit of 15–25 mm, based on the core size as seen on (DWI) MRI, our study focused on the smaller infarcts, and thus more difficult to detect compared to other studies where the upper size limit ranged from 15 to 25 mm.^1^, ^2^, ^7^ The filters were optimized to the smallest ALI size of 5 mm by setting σ_d_ equal to 2 mm leading to more contrast but also a higher noise level in the CBV maps than desired.

In addition, as the ALIs were defined as tissue without any perfusion and no surrounding penumbra, we chose to use the CBV map instead of additional parameter maps such as cerebral blood flow (CBF), mean‐transit‐time (MTT), and time‐to‐peak (TTP). This may have limited our findings. A systematic review showed that, although clinical CTP has a general low sensitivity for lacunar stroke detection, several studies consider TTP and MTT to be more sensitive in lacunar stroke detection than CBV.^7^ Further studies should include the different CTP parameter maps in ALI evaluation.

Another drawback of the dataset is the used tube voltage. Scanning at 120 kVp and 35 mAs leads to less iodine‐induced contrast enhancement and higher noise compared to common settings around 80 kVp and 200 mAs.

## 5 CONCLUSIONS

The findings of this study with artificial lacunar infarcts underscore the superiority of the temporal‐average guided filter over TIPS and U‐Net for denoising thin‐slice CTP images and the added value of thin‐slice reconstructions. The enhanced resilience to noise and benefit of increased spatial resolution is reflected in an improved CNR, observer confidence, and overall detection accuracy, thereby enhancing the identification of lacunar strokes in CTP imaging. These findings will have to be confirmed by detection of true lacunar infarcts in a clinical setting of acute stroke.

## CONFLICT OF INTEREST STATEMENT

The authors have no relevant conflicts of interest to disclose.

## References

[1] Arba F , Mair G , Phillips S , Sandercock P , Wardlaw JM , Third International Stroke Trial Collaborators . Improving clinical detection of acute lacunar stroke: analysis from the IST‐3. Stroke. 2020;51(5):1411‐1418. doi:10.1161/STROKEAHA.119.028402 32268853 PMC7185055

[2] Regenhardt RW , Das AS , Lo EH , Caplan LR . Advances in lacunar stroke pathophysiology: a review. JAMA Neurol. 2018;75(10):1273‐1281. doi:10.1001/jamaneurol.2018.1073 30167649 PMC7426021

[3] Yaghi S , Raz E , Yang D , et al. Lacunar stroke: mechanisms and therapeutic implications. J Neurol Neurosurg Psychiatry. doi:10.1136/jnnp-2021-326308. Published online May 26, 2021:jnnp‐2021‐326308. 34039632

[4] Gore M , Bansal K , Khan Suheb MZ , Lui F , Asuncion RMD . Lacunar stroke. StatPearls. StatPearls Publishing; 2025. Accessed May 7, 2025. http://www.ncbi.nlm.nih.gov/books/NBK563216/ 33085363

[5] Biesbroek JM , Niesten JM , Dankbaar JW , et al. Diagnostic accuracy of CT perfusion imaging for detecting acute ischemic stroke: a systematic review and meta‐analysis. Cerebrovasc Dis. 2013;35(6):493‐501. doi:10.1159/000350200 23736122 10.1159/000350200

[6] Konstas AA , Goldmakher GV , Lee TY , Lev MH . Theoretic basis and technical implementations of CT perfusion in acute ischemic stroke, part 1: theoretic basis. AJNR Am J Neuroradiol. 2009;30(4):662‐668. doi:10.3174/ajnr.A1487 19270105 10.3174/ajnr.A1487 PMC7051780

[7] Zedde M , Napoli M , Grisendi I , et al. CT perfusion in lacunar stroke: a systematic review. Diagnostics (Basel). 2023;13(9):1564. doi:10.3390/diagnostics13091564 37174955 10.3390/diagnostics13091564 PMC10177869

[8] Wu D , Ren H , Li Q , Self‐supervised dynamic CT perfusion image denoising with deep neural networks. Published online May 19, 2020. doi:10.48550/arXiv.2005.09766

[9] Mendrik AM , Vonken EJ , van Ginneken B , et al. TIPS bilateral noise reduction in 4D CT perfusion scans produces high‐quality cerebral blood flow maps. Phys Med Biol. 2011;56(13):3857‐3872. doi:10.1088/0031‐9155/56/13/008 21654042 10.1088/0031-9155/56/13/008

[10] Tomasi C , Manduchi R , Bilateral filtering for gray and color images. In: Sixth International Conference on Computer Vision (IEEE Cat. No.98CH36271) . Narosa Publishing House; 1998:839‐846. doi:10.1109/ICCV.1998.710815

[11] Kauw F , van Ommen F , Bennink E , et al. Early detection of small volume stroke and thromboembolic sources with computed tomography: rationale and design of the ENCLOSE study. Eur Stroke J. 2020;5(4):432‐440. doi:10.1177/2396987320966420 33598562 10.1177/2396987320966420 PMC7856586

[12] Kauw F , Velthuis BK , Takx RAP , et al. Detection of cardioembolic sources with nongated cardiac computed tomography angiography in acute stroke: results from the ENCLOSE study. Stroke. 2023;54(3):821‐830. doi:10.1161/STROKEAHA.122.041018 36779342 10.1161/STROKEAHA.122.041018 PMC9951793

[13] Metz CT , Klein S , Schaap M , van Walsum T , Niessen WJ . Nonrigid registration of dynamic medical imaging data using nD + t B‐splines and a groupwise optimization approach. Med Image Anal. 2011;15(2):238‐249. doi:10.1016/j.media.2010.10.003 21075672 10.1016/j.media.2010.10.003

[14] Bennink E , Oosterbroek J , Kudo K , Viergever MA , Velthuis BK , de Jong HWAM . Fast nonlinear regression method for CT brain perfusion analysis. J Med Imaging (Bellingham). 2016;3(2):026003. doi:10.1117/1.JMI.3.2.026003 27413770 10.1117/1.JMI.3.2.026003 PMC4918691

[15] Ronneberger O , Fischer P , Brox T , U‐Net: convolutional networks for biomedical image segmentation. Published online May 18, 2015. doi:10.48550/arXiv.1505.04597

[16] Lehtinen J , Munkberg J , Hasselgren J , et al. Noise2Noise: learning image restoration without clean data. Published online October 29, 2018. doi:10.48550/arXiv.1803.04189

[17] Cereda CW , Christensen S , Campbell BCV , et al. A benchmarking tool to evaluate computer tomography perfusion infarct core predictions against a DWI standard. J Cereb Blood Flow Metab. 2016;36(10):1780‐1789. doi:10.1177/0271678x15610586 26661203 10.1177/0271678X15610586 PMC5076783

[18] Hakim A , Christensen S , Winzeck S , et al. Predicting infarct core from computed tomography perfusion in acute ischemia with machine learning: lessons from the ISLES challenge. Stroke. 2021;52(7):2328‐2337. doi:10.1161/STROKEAHA.120.030696 33957774 10.1161/STROKEAHA.120.030696 PMC8240494

[19] Yushkevich PA , Piven J , Hazlett HC , et al. User‐guided 3D active contour segmentation of anatomical structures: significantly improved efficiency and reliability. NeuroImage. 2006;31(3):1116‐1128. doi:10.1016/j.neuroimage.2006.01.015 16545965 10.1016/j.neuroimage.2006.01.015

[20] Förster A , Kerl HU , Wenz H , Brockmann MA , Nölte I , Groden C . Diffusion‐ and perfusion‐weighted imaging in acute lacunar infarction: is there a mismatch?. PLOS ONE. 2013;8(10):e77428. doi:10.1371/journal.pone.0077428 24130885 10.1371/journal.pone.0077428 PMC3795042

